# Research on the Compound Optimization Method of the Electrical and Thermal Properties of SiC/EP Composite Insulating Material

**DOI:** 10.3390/polym13193369

**Published:** 2021-09-30

**Authors:** Xupeng Song, Xiaofeng Xue, Wen Qi, Jin Zhang, Yang Zhou, Wei Yang, Yiran Zhang, Boyang Shen, Jun Lin, Xingming Bian

**Affiliations:** 1 State Key Laboratory of Alternate Electrical Power System with Renewable Energy Sources, North China Electric Power University, Beijing 102206, China; songxupeng96@163.com (X.S.); xxf7002@163.com (X.X.); qiwen0602@163.com (W.Q.); Jun.lin@ncepu.edu.cn (J.L.); 2 State Grid Corporation of China, Co., Ltd., Beijing 100031, China; jin-zhang@sgcc.com.cn (J.Z.); yang-zhou@sgcc.com.cn (Y.Z.); 3 State Key Laboratory of Advanced Power Transmission Technology, Global Energy Interconnection Research Institute, Co., Ltd., Beijing 102211, China; 19630100@163.com; 4 State Grid Hubei Maintenance Company Yichang Operation Maintenance Branch, Yichang 430050, China; yr495459435@163.com; 5 Electrical Engineering Division, University of Cambridge, Cambridge CB3 0FA, UK; bs506@cam.ac.uk

**Keywords:** SiC crystal form, micro-nano compound, thermal conductivity, breakdown field strength

## Abstract

In this paper, in order to improve the electrical and thermal properties of SiC/EP composites, the methods of compounding different crystalline SiC and micro-nano SiC particles are used to optimize them. Under different compound ratios, the thermal conductivity and breakdown voltage parameters of the composite material were investigated. It was found that for the SiC/EP composite materials of different crystal types of SiC, when the ratio of α and β silicon carbide is 1:1, the electrical performance of the composite material is the best, and the breakdown strength can be increased by more than 10% compared with the composite material filled with single crystal particles. For micro-nano compound SiC/EP composites, different total filling amounts of SiC correspond to different optimal ratios of micro/nano particles. At the optimal ratio, the introduction of nanoparticles can increase the breakdown strength of the composite material by more than 10%. Compared with the compound of different crystalline SiC, the advantage is that the introduction of a small amount of nanoparticles can play a strong role in enhancing the break-down field strength. For the filled composite materials, the thermal conductivity mainly depends on whether an effective heat conduction channel can be constructed. Through experiments and finite element simulation calculations, it is found that the filler shape and particle size have a greater impact on the thermal conductivity of the composite material, when the filler shape is rounder, the composite material can more effectively construct the heat conduction channel.

## 1. Introduction

The filled high thermal conductivity composite material is to dope inorganic fillers or metal fillers with high thermal conductivity into the polymer matrix to build a thermally conductive network. Filled thermally conductive polymers are simple to operate, low in cost, suitable for industrial production and have become the mainstream development direction of high thermally conductive insulating materials. [1,2,3,4,5,6,7].

The type, distribution and filling amount of the fillers incorporated have a great impact on the electrical and thermal properties of the composite material [8,9,10,11]. Many studies modified the epoxy matrix by introducing two or more types of powder fillers into the resin [12,13,14,15]. For example, Zhou et al. [16] added 6 wt% multi-walled carbon nanotubes (MWCNT) or 71.7 wt% micron-sized silicon carbide (SiC) to the epoxy resin to maximize the thermal conductivity of the composites, and then partially replaced the micron-sized fillers with nano-sized fillers. This paper took the advantage of the large aspect ratio of one-dimensional structure of MWCNTs, making MWCNTs effectively act as thermal conductivity channels between micron SiC particles and form a more effective three-dimensional infiltration network for heat flow. Zhao et al. [17] used two-dimensional boron nitride nanosheets (BNNS) and zero-dimensional boron nitride microspheres (BNMS) to build a three-dimensional thermal conductivity network, which can effectively improve the thermal conductivity of epoxy composites. Tu et al. [18] studied the surface charge transfer characteristics and the control mechanism of the coating to illustrate the influence of the surface coating on the surface charge accumulation characteristics of epoxy resin. The surface charge of epoxy resin can be effectively suppressed by coating 1-3 wt% micron SiO_2_ particles or 3 wt% nano SiO_2_ particles. Takahiro et al. [19] prepared micro-nano epoxy composites by dispersing nano-layered silicate filler and micron silica (SiO_2_) filler in the epoxy matrix. Compared with the conventional SiO_2_ filling, the internal structure of the composite obtained by micro-nano composite was tighter, and the breakdown strength of the composite was 7% higher than that of the conventional filled epoxy composite. Huang et al. [20] prepared alumina (Al_2_O_3_)/silicon carbide (SiC)/epoxy composites by sol-gel method. The results showed that the introduction of dense and uniform Al_2_O_3_ on the SiC surface improved the interface adhesion between the epoxy matrix and SiC particles, and the thermal resistance of the filler-matrix interface was reduced. Wu J [21] et al. magnetized the SiC particles so that the paramagnetic SiC particles were aligned along the magnetic field lines under the action of an external magnetic field. After thermosetting, an ordered epoxy/silicon carbide composite material was obtained, and the relationship between the distribution state of silicon carbide and the dielectric properties and thermal conductivity of the composite material was explored. Seenaa Hussein [22] et al. studied the modification method of poly(vinyl alcohol) (PVA)/poly(vinyl pyrrolidone) (PVP) polymer film. By using a solution blending method to incorporate nano-graphene fillers into PVP/PVA, the thermal conductivity and mechanical properties of the composite material are greatly improved. At the same time, as the graphene content increases, the thermal weight loss rate of the composite material decreases, which proves that there is a strong interface interaction between the nano-graphene and the matrix. Alaa M. Abd-Elnaiem [23] et al. used sol-gel polymerization method to coat multi-walled carbon nanotubes (MWCNTs) with tetraethyl orthosilicate (TEOS) to form a covalent bond with the epoxy resin matrix when used as a filler to enhance the interface effect. Epoxy-TEOS/MWCNTs composites filled with different proportions were prepared. When the filling ratio was 4 wt%, the mechanical, optical and thermal properties of the epoxy composites reached the best. Nadia A [24] et al. prepared polymethyl methacrylate (PMMA)/MWCNTs composites using a solution blending method. The covalent bond and hydrogen bond between PMMA and MWCNTs were analyzed by infrared spectroscopy, which proved the strong interaction between the filler and the matrix. By adding an appropriate proportion of MWCNTs filler, the mechanical properties and electrical conductivity of the composite material have been greatly improved. Seenaa I. Hussein [25] et al. used graphene, carbon nanotubes and carbon fibers as fillers, respectively, to fabricate epoxy resin-based composite materials. Under the action of the filler, the wear rate of the composite material is significantly reduced, while the thermal conductivity is greatly improved. Among them, graphene and carbon nanotubes have the most significant increase in the thermal conductivity of composite materials.

The SiC-filled composite dielectric has nonlinear conductivity characteristics [26,27]. When the local electric field in the insulating medium is distorted, it can homogenize the electric field [28,29]. At the same time, filling with SiC can significantly improve the thermal conductivity of the composite material, so SiC/EP has broad application prospects in the packaging of high-capacity power devices and the insulation of high-voltage equipment. However, the introduction of SiC will decrease the breakdown strength of composite materials [30]. In the current research on SiC/EP composite materials, attention has been paid to the electrical and thermal properties of the composite materials filled with various crystal forms of SiC, but there is still a lack of relevant research on the combined effects of different crystal forms. In view of the above problems, this paper studies the effects of different crystalline SiC compound and micro-nano particle compound on the breakdown field strength and thermal conductivity of SiC/EP composites. Suggestions for improving the electrical and thermal properties of SiC/EP composites are provided from the perspective of particle compounding.

## 2. Materials and Methods

### 2.1. Materials

Bisphenol A epoxy resin was used as the matrix. This kind of epoxy resin can fuse well with many additives, with excellent bonding strength and strong corrosion resistance. Anhydride curing agent has a good mixing property with epoxy, the viscosity of the mixed liquid is low, which is convenient for the mixing of filler particles, and the electrical and mechanical properties of the cured epoxy composites are improved to a certain extent. Silicon carbide (SiC) with different crystal shape and particle size was selected as filler. In order to improve the binding degree of nano-SiC particles and epoxy resin and enhance the dispersion of filler particles, the surface modification of nano-SiC particles was carried out by using silane coupling agent (KH-560). The raw materials used in the experiment are listed in Table 1.

### 2.2. Sample Preparation

**Figure 1:**
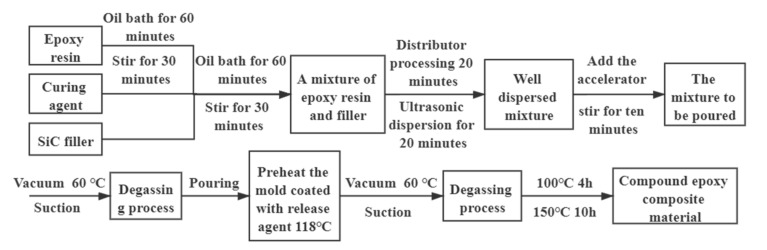
Epoxy composite preparation process.

In order to study the effect of different crystalline SiC composite and micro/nano SiC composite on the electrical and thermal characteristics of the material. Under the premise that the overall filling ratio remains unchanged, we changed the proportions of different crystalline SiC and micro/nano SiC particles in the filler. Since the physical and chemical properties of silicon carbide and epoxy resin are different, the particles in epoxy are easy to agglomerate. However, the silicon carbide should evenly disperse in the matrix as far as possible. The flow chart is shown in Figure 1, and the specific experimental steps are as follows:

(1) The required mass of epoxy resin was weighed and poured into a three-port flask. The oil bath was heated to 60 °C to improve the fluidity of the matrix, and the water vapor adsorbed by the epoxy was mechanically stirred for 30 min to discharge the water vapor absorbed by the epoxy during storage.

(2) With the mass ratio of epoxy: curing agent of 100:85, the curing agent was weighed into the epoxy, and the silicon carbide filler particles were weighed according to the corresponding ratio. After full grinding, the epoxy curing agent mixed solution was added, and the mixture was mixed in the oil bath at 60 °C at a constant speed of 360 r/min for 60min to ensure uniform distribution of filler particles.

(3) After the matrix and the filler were fully mixed, the matrix was dispersed by ultrasonic for 20 min, and the high-speed disperser was treated for 20 min. The mass ratio of epoxy:accelerator = 100:1 drop and the accelerator were added, and the mixture was mixed at 260 r/min at a constant speed for 10min.

(4) Pour out the mixed solution and put it in a vacuum drying box, keep it at 60 °C for vacuum operation, and extract it for many times until no obvious bubbles overflow.

(5) Put the mold sprayed with release agent into the blast dryer for preheating, and the temperature was 100 °C for 30 min. Pour the vacuum pumped mixed solution into the mold and put it in the vacuum oven for degassing again. The temperature was 60 °C for 30 min. The silicon carbide epoxy composite was obtained.

When the filling ratio of filler particles reaches 20 vol%, the thermal conductivity of the composite material is significantly improved, but when the filling ratio exceeds 30 vol%, the liquid mixture is very viscous, and it is difficult to vacuum out the internal bubbles and cast molding. Therefore, we chose the most representative 20 vol% and 30vol% as the total filling amount, which have better comprehensive performance. The filling ratios of different filler are shown in Table 2 and Table 3:

In order to improve the bonding degree between the surface of SiC particles and epoxy resin matrix, the silane coupling agent KH-560 was used to organically modify the surface of SiC particles [7]. The modification method was as follows:

(1) According to the volume ratio of ethanol and ultra-pure water of 19:1, 400 mL solution was mixed and poured into the flask. 50 g SiC particles were weighed and dried at 80 °C for 6 h and added into the flask. The SiC particles were stirred at 60 °C for 10 min to fully disperse in the solvent;

(2) 2.5 g silane coupling agent KH-560 was dropped into SiC/ethanol/aqueous solution and stirred at 60 °C for 6h to fully react.

(3) Pour the solution into the plastic reagent bottle, use centrifuge to separate SiC particles and solvent, take out after drying at 50 °C for 8 h, and grind with agate mortar for reserve.

### 2.3. Material Characterization and Testing

**Figure 2:**
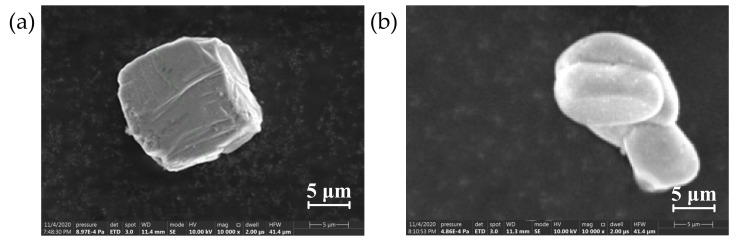
Electron microscope of SiC particles with different crystal types. (**a**) 10 µm α-SiC; (**b**) 10 µm β-SiC.

**Figure 3:**
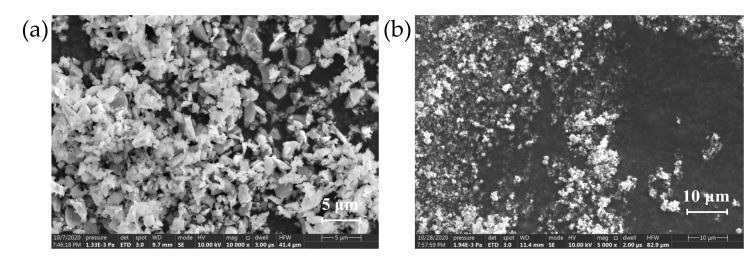
Electron microscopy of micron and nanometer SiC particles. (**a**) 1.5 µm β-SiC; (**b**) 50 nm β-SiC.

Scanning electron microscope (SEM) Quanta FEG 250 was used to observe α-SiC and β-SiC packing particles with different crystal types. α-SiC has hexagonal crystal structure, and its surface morphology is more distinct, β-SiC is cubic crystal, and its surface morphology is rounder. The SEM of SiC particles are shown in Figure 2 and Figure 3.

Transient plane heat source method was used to measure the thermal conductivity of the material using the thermal conductivity meter TPS 2500S. The sample used for the test was a wafer with the radius of 35 mm and thickness of 3 mm. In the room temperature, the breakdown strength tester HCDJC-50kV was used for the sample breakdown test. The voltage was increased at a rate of 2 kV/s until electric breakdown occurred. The samples used to measure the breakdown voltage were discs with a thickness of 1 mm and a diameter of 50 mm. 10 samples were tested for each group of materials.

## 3. Electrical and Thermal Properties of Epoxy Resin Composite with Different Crystal Forms of SiC

Different crystal types of SiC have different atomic packings, so their macroscopic electrical and thermal properties are different. By preparing epoxy resin composite materials with different crystal forms of SiC, and testing their electrical and thermal properties, the influence of the interaction between the filler and matrix on the properties of the composite materials was explored.

### 3.1. Breakdown Strength Analysis of Composite Materials

Two-parameter Weibull distribution was used to analyze the breakdown data of SiC/EP composites. The equation of two-parameter Weibull distribution is as follows:

(1) $$F(x)=1-\exp\left(-{\left(\frac{x}{\alpha }\right)}^{\beta }\right)$$

where *x* is a variable, representing the breakdown data of the sample obtained by the test; *β* is the shape parameter, the dispersion of the data decreases with the increase of the value; *α* is the scale parameter, representing the breakdown strength when the breakdown probability is 63.2%, which is also called the average breakdown strength.

The least square method and Ross failure probability distribution function were used to solve the correlation coefficient, and the critical value criterion of the correlation coefficient was used to judge whether the breakdown data complied with the two-parameter Weibull distribution.

Ross failure probability distribution function is:

(2) $$F\left(i,n\right)=\frac{i-0.44}{n+0.25}\times 100\%$$

The experimental breakdown voltage data were arranged according to their size and processed by formula. Least square method for linear fitting was used to solve the correlation coefficient.

(3) $$X_{i}=\ln\left(E_{i}\right)$$

(4) $$Y_{i}=\ln\left\{-\ln\left(1-\frac{F\left(i,n\right)}{100}\right)\right\}$$

**Figure 4:**
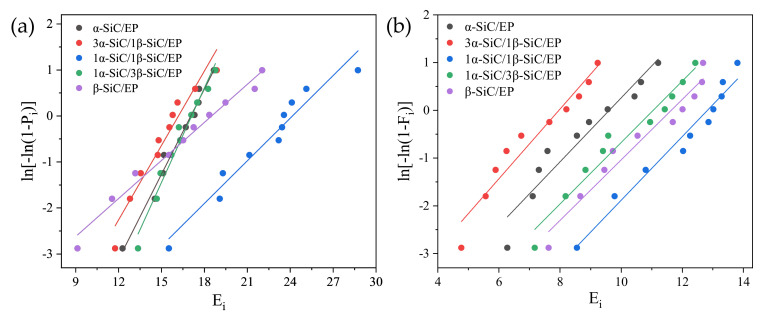
Weibull distribution of composite material breakdown strength. (**a**) 20 vol% SiC/EP; (**b**) 30 vol% SiC/EP.

As shown in Figure 4, the data processing results showed that the breakdown strength data of SiC/EP composites conformed to Weibull distribution.

**Figure 5:**
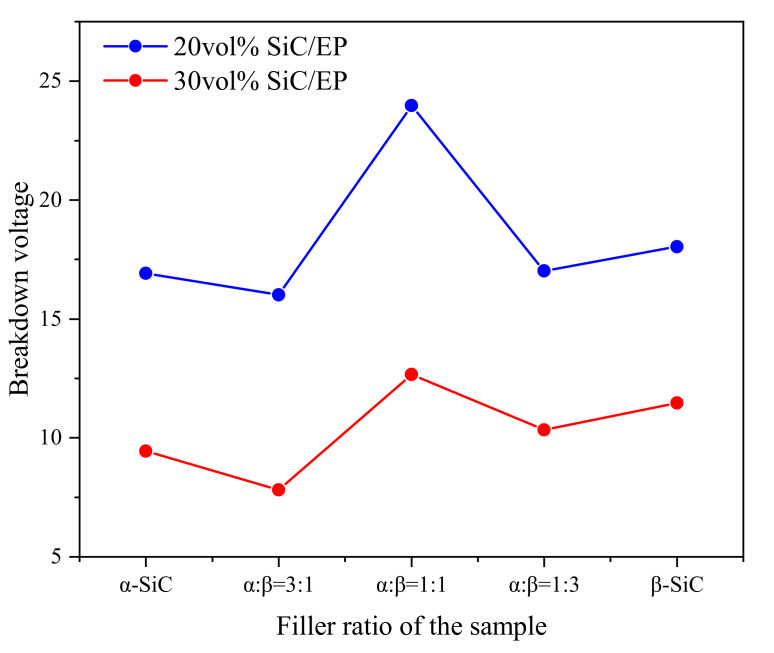
Average breakdown strength of composite material.

By comparing the two groups of composites with different filling quantities in Figure 4, the breakdown strength showed a consistent trend with the change of the ratio of the two crystal forms. The breakdown strength of β-SiC composite is higher than α-SiC. When the ratio of α-SiC to β-SiC is 3:1 or 1:3, the breakdown strength is significantly lower than the composite filled with single crystal SiC. When the ratio of α-SiC to β-SiC is 1:1, the breakdown strength is the highest. It can be seen in Figure 5 that when the total filling ratio is 20vol%, the breakdown field strength of the composite material with a ratio of α-SiC:β-SiC of 1:1 is increased by 41.7% compared with single α-SiC crystal type filling, and 32.9% compared with single β-SiC crystal type filling. When the total filling ratio is 30 vol%, compared with the single α-SiC crystal type filling, the breakdown field strength of the composite material with a ratio of α-SiC:β-SiC of 1:1 is increased by 34.0%, and compared with the single β-SiC crystal type filling, it is increased by 10.4%.

It can be seen from the SEM images of two different crystal forms of SiC that the morphologies of the two fillers are not completely regular, the shape of α-SiC is relatively sharp, and the surface of β-SiC is comparatively round. When the composite material uses two different crystal forms of SiC as the filler of the composite, it will lead to internal defects of the material, which results in a significant decrease in the breakdown strength of the composite material. When the compounding ratio is 1:1, the internal defects of the material are obviously reduced, and the bonding degree of the interface between the filler and the matrix is obviously improved. The breakdown path cannot form along the air gap defect and the development path extend, which improves the breakdown strength of the material to a certain extent. This conjecture was verified by analyzing the infrared spectrum.

**Figure 6:**
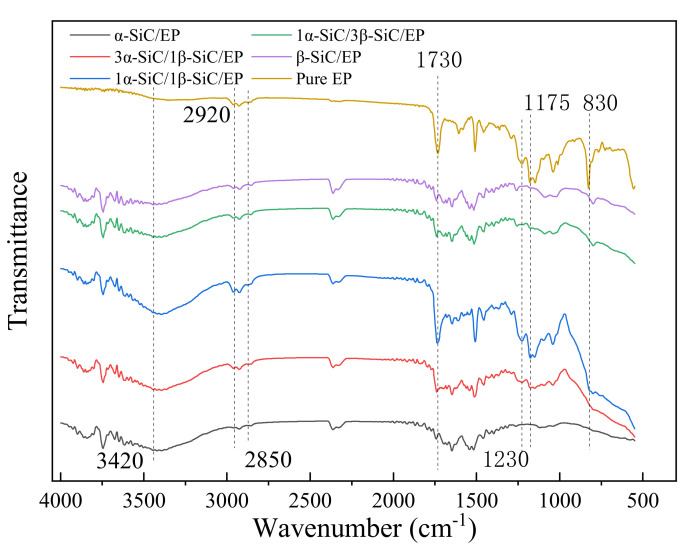
Infrared spectrum of SiC/EP composite material.

**Figure 7:**
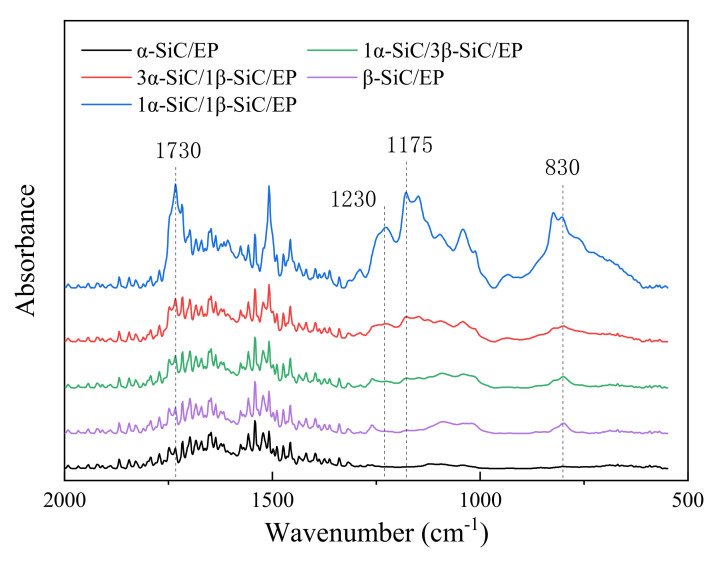
Partial enlarged view of baseline correction of different crystal SiC/EP infrared spectra.

In order to compare the relative size of each absorption peak more intuitively and quantitatively, the infrared absorption spectrum was converted into a projection spectrum, and the method of baseline correction was adopted to make the spectral values at both ends of each absorption peak lying on a straight line. Enlarge the characteristic peak area, as shown in Figure 6 and Figure 7.

The wave numbers of 1230 cm^−1^ and 1170 cm^−1^ are the absorption bands of the ester [31,32,33]. The wave number of 2850cm^−1^ represents the symmetrical stretching vibration of C-H in CH3 [24]. The wave number of 2920cm^−1^ is attributable to the vibration of aromatic protons, and the broad peak at 3420cm^−1^ is hydroxyl and hydrogen bonds [23]. By comparing the infrared spectra of the five formula systems, it can be found that the crosslinking degree of epoxy resin composite material has decreased to varying degrees compared with the pure epoxy resin, which also reflects the reduction of the breakdown strength of the composite material. A higher degree of cross-linking indicates that the epoxy resin molecules are more closely linked, and the defects between the insulating material and the filler in the system are reduced, the breakdown path is difficult to develop along the interface [34,35], which increases the breakdown strength of the composite material. For the five composite material systems, when the volume ratio of α-SiC to β-SiC was 1:1, the crosslinking degree of the composite material was the highest, so it also had a higher breakdown strength. This may be due to the different crystal forms of α-SiC and β-SiC and the better overlap between the particles, which reduced the agglomeration of the same crystal particles and played a certain mutual coordination during the condensation reaction, so as to improve the crosslinking degree of epoxy

Compared with 20 vol% composite materials, the overall breakdown strength of composite materials filled with 30 vol% was reduced. As a crystal with high electrical conductivity, SiC has high breakdown strength, therefore, it is the epoxy resin matrix that determines the breakdown strength of the composite material. When the SiC filling amount increased, due to the micro-particles and the matrix interface were not tightly bonded, the internal air gap defects greatly increased, the development path of the electrical tree in the epoxy was shortened, and the breakdown strength was significantly reduced [36,37]. When other inorganic fillers such as SiO_2_ and BN are used [38,39], the breakdown field strength of the composite material exhibits consistent characteristics at high filling ratios, and this trend is not conducive to the application of thermally conductive insulating materials.

**Figure 8:**
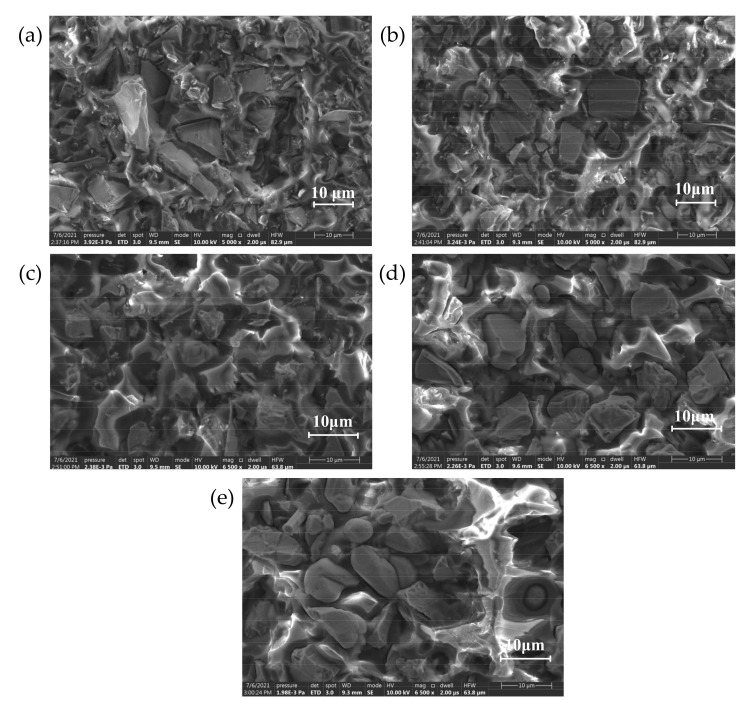
Sectional electron micrograph of SiC/EP composite material. (**a**) α-SiC/EP; (**b**) α-SiC:β-SiC = 3:1; (**c**) α-SiC:β-SiC = 1:1; (**d**) α-SiC:β-SiC = 1:3; (**e**) β-SiC/EP.

In order to investigate the effect of crystal configuration on the crosslinking degree in terms of microstructure, the cross sections of the composites with different crystal configuration were observed by scanning electron microscopy. The focus was on the state of the interface between the filler particles and the matrix and the dispersibility of the filler particles. The SEM image is shown in Figure 8:

It can be seen from the microscopic morphology that the filler particles are uniformly distributed in the matrix without delamination or particle aggregation. In the filling system, α-SiC particles have more edges and corners, while the shape of β-SiC is more rounded, and there are obvious air gap defects at the interface between the filler particles and the matrix. When the ratio of α-SiC to β-SiC was 1:1, the bonding degree between the filler and the matrix was better and the defects at the interface were significantly reduced. There are no protruding particles or obvious pits at the cross-section, which appears to be compact structure. This shows that the two crystal particles overlap and cooperate with each other in the mixing and solidification process under the appropriate ratio, which can improve the uniformity of the mixed system. The degree of cross-linking of epoxy resin between particles is increased, which significantly reduces defects at the interface of the composite material [40].

### 3.2. Thermal Conductivity of SiC/EP Composites

**Figure 9:**
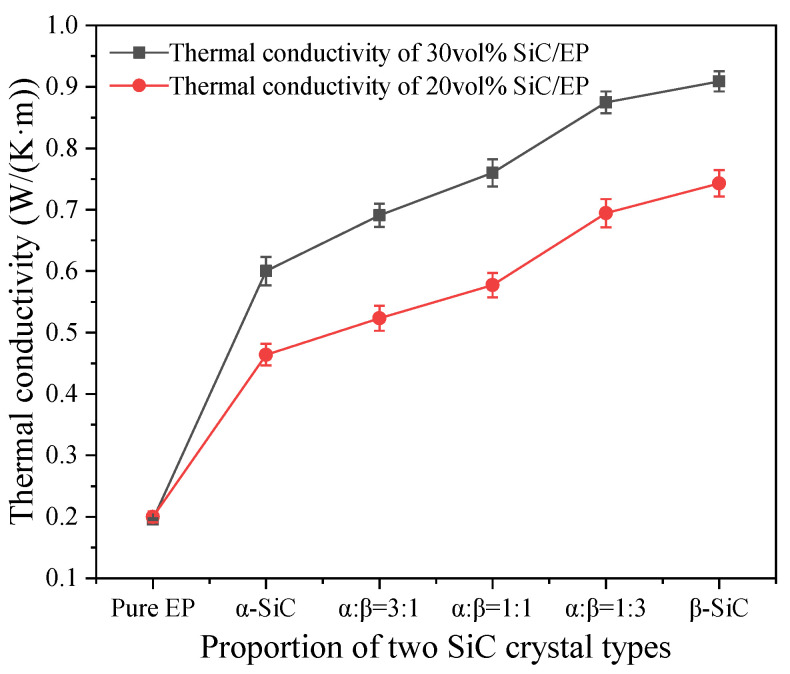
Thermal conductivity of SiC composite filled with different crystal forms.

The Figure 9 shows the variation of thermal conductivity of two crystal SiC composites in five proportions. Since the epoxy resin has low cross-linking crystallinity and disorder, only the thermal motion of macromolecular chains and groups cannot provide a way for the rapid movement of phonons, so the thermal conductivity of pure epoxy resin material is only 0.196 W/(m·K). The thermal conductivity of β-SiC composite was significantly higher than α-SiC composite when the volume fraction of β-SiC was fixed and the ratio of two different crystal forms was changed. The thermal conductivity of β-SiC/epoxy composites was 0.7433 W/(m·K) and 0.9019 W/(m·K), respectively, which was 61.13% and 48.58% higher than α-SiC/epoxy composites with the same amount of filling, and 279.23% and 360.15% higher than pure epoxy resin. 

The thermal conductivity of the composite material of the two crystal forms increased with the increasing of the proportion of β-SiC. At present, the thermal path theory is the most common theory used to explain the thermal conduction mechanism of filled thermally conductive composites. This theory describes that the high thermal conductivity filler particles in the matrix overlap each other to form a thermal conduction path, and the heat flux is transferred from the high temperature direction to the low temperature along the network built by the thermal conductive filler [41,42]. The results showed that the crosslinking degree of epoxy matrix had little effect on the thermal conductivity of filled composites, but the shape of filler had much effect on the thermal conductivity of filled composites. Compared with α-SiC, β-SiC particles have more rounded shapes, better bonding degree with epoxy resin matrix, and lower interface thermal resistance. Furthermore, β-SiC particles have more effective contact area between particles than those with sharp edges and angles, which is more conducive to the formation of thermal conductivity channels.

### 3.3. Simulation Study on the Influence of Different Shapes of SiC Particles on the Thermal Conductivity of Composites

In order to study the influence of the shape of filler particles on the construction of the internal heat conduction channel of the composite material, the finite element method [5,43,44] was used to construct a solid heat transfer model for the internal representative volume elements (RVE) of the filled composite material. In the steady state, according to Fourier’s law of heat conduction, the differential equation for solving the internal heat conduction of the composite material was obtained, as in the following equation:

(5) $$\frac{\partial^{2}T}{\partial^{2}x}+\frac{\partial^{2}T}{\partial^{2}y}+\frac{\partial^{2}T}{\partial^{2}z}=0$$

We set the side surface of the composite material as thermal insulation, which was also the second type of boundary condition, as in the following formula:

(6) $${\left.\frac{\partial T}{\partial n}\right|}_{\Gamma }=0$$

In the formula, the subscript Γ denoted the side surface of the composite material. Set the initial temperature on the upper surface (S_1_) to 60 °C, and set the temperature on the lower surface (S_2_) to 20 °C, which was also the first type of boundary condition, as in the following formula:

(7) $${\left.T\right|}_{s_{1}}=60{}^{\circ}C;\;{\left.T\right|}_{s_{2}}=20{}^{\circ}C$$

**Figure 10:**
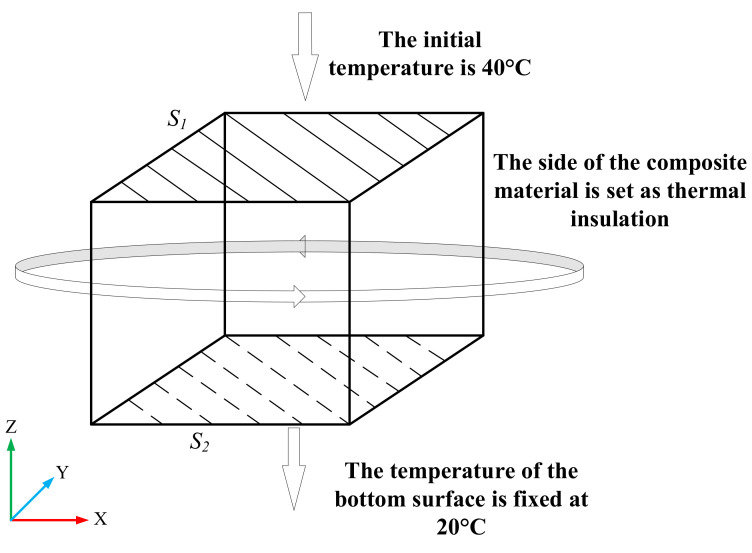
Model boundary settings.

The setting of boundary conditions is shown in Figure 10.

Using the finite element method, by solving the heat conduction equation (1), the conduction heat flux inside the composite material was obtained. According to the Fourier law of heat conduction, the thermal conductivity of the composite material was calculated by the following formula [45]:

(8) $$K=\frac{QL}{\Delta T}$$

In the formula, *K* is the equivalent thermal conductivity; *Q* is the average conductive heat flux in the z-direction inside the composite material; *L* is the thickness of the model in the *z*-axis direction; Δ*T* is the temperature difference between the upper surface and the bottom surface in the *z*-axis direction.

**Figure 11:**
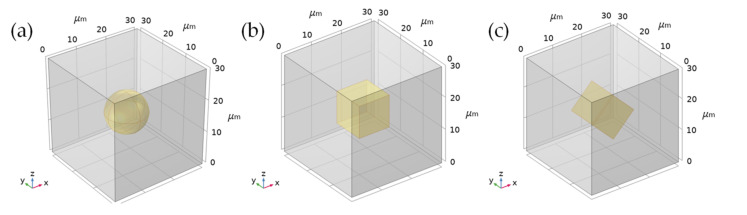
The geometric model of a composite material filled with a single particle. (**a**) A single spherical particle; (**b**) A single cubic particle placed horizontally; (**c**) A single cubic particle rotated 45°.

In order to study the influence of particle shape on the heat flux of the surrounding matrix, a single particle was placed in a cube RVE with a side length of 30 μm. Spherical and cubic particles of the same size were placed, and the cubes were placed in parallel and rotated 45° to make the calculation results representative. As shown in Figure 11.

**Figure 12:**
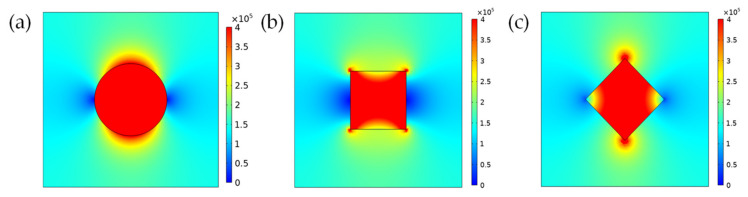
Cross-section conductive heat flux cloud diagram of composite materials. (**a**) Filler particle is small sphere; (**b**) Filler particle is horizontally placed cube; (**c**) Filler particle cube is rotated 45°.

**Figure 13:**
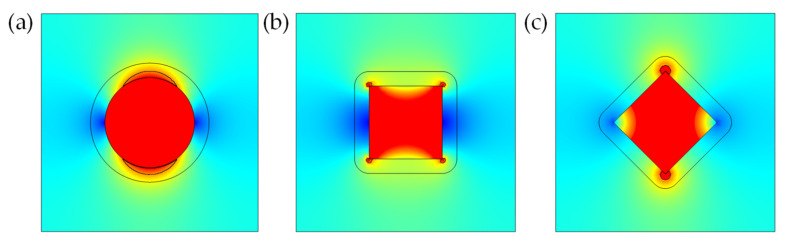
Cloud iamge of conductive heat flux after ImageJ processing. (**a**) Filler particle is small sphere; (**b**) Filler particle is horizontally placed cube; (**c**) Filler particle cube is rotated 45°.

Taking the cross section parallel to the *xz* plane and *y* = 15 μm to study the conductive heat flux of the filler and its surrounding matrix, as shown in Figure 12:

Considering that the filler particles construct a heat conduction channel through effective contact, it is believed that when the conductive heat flux of the matrix between the fillers reaches 80% of the filler [5], the matrix between the fillers can participate in the construction of an effective heat conduction channel. We used the ImageJ software to process the conduction heat flux cloud image. Taking the conduction heat flux at the center of the filler particles as the reference, the conduction heat flux at the center of the particle was red in the chromatogram, and the corresponding color gamut value was 0; the point where the conduction heat flux was 0 is blue in the chromatogram, and the corresponding color gamut value was 158. When the conductive heat flux in the matrix was 80–100% of the heat flux in the center of the particle, the color gamut value range of the matrix in the three cases can be obtained by calculation, and the area within this range was integrated in ImageJ, as shown in Figure 13:

The effective contact areas obtained in the three cases were: (a) 9.941, (b) 1.34 and (c) 2.405. It can be seen that when the filler is spherical particles, because the surface was more rounded, according to the principle of heat transfer, the effective heat transfer area of the surrounding matrix was larger, while the effective heat transfer area of the matrix surrounding the cubic particles was much smaller.

In order to study the influence of multiple particles on the thermal conductivity of composite materials, a three-dimensional random particle distribution model was established. We took the composite material RVE as a cube with a side length of 50 μm, and filled it with 20 vol% particles, corresponding to the composite material of different crystal types, and the numbers of particles were set as shown in Table 4.

**Figure 14:**
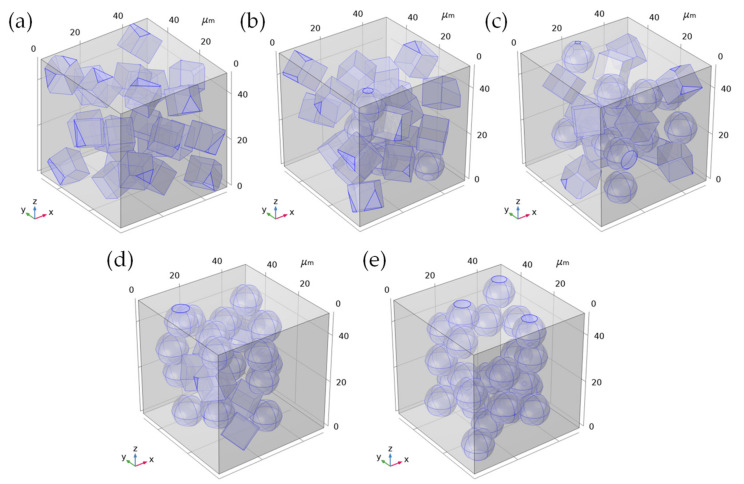
RVE geometric model of randomly filled composite materials with particles. (**a**) Model corresponding to β/EP; (**b**) Model corresponding to 1α/3β/EP; (**c**) Model corresponding to 1α/1β/EP; (**d**) Model corresponding to 3α/1β/EP; (**e**) Model corresponding to α/EP.

**Figure 15:**
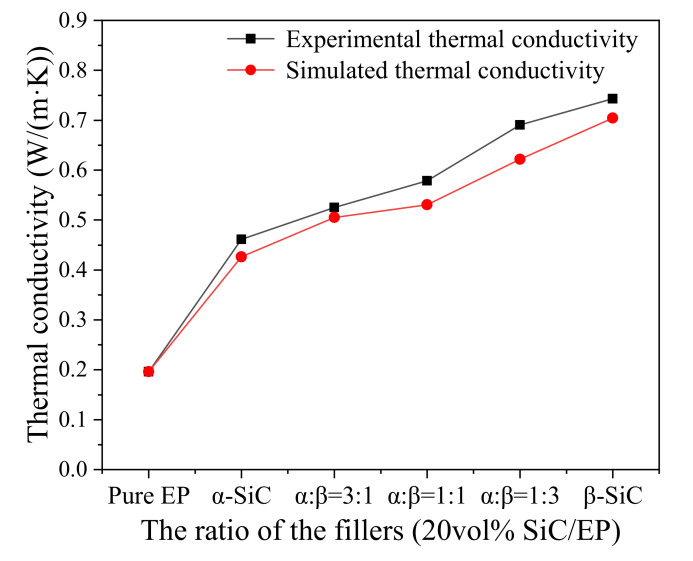
Comparison of experimental and simulated composite thermal conductivity results.

The established RVE model is shown in Figure 14:

Comparing the thermal conductivity of the volume representative element (RVE) obtained by the simulation with the experimental results is shown in the Figure 15. The results show that the finite element simulation can be consistent with the experimental results. Therefore, the simulation results can be used to explain the influence of the shape of the filler particles on the heat of the composite material. As the proportion of spherical particles increases, the thermal conductivity of the composite material increases. This is due to the effective heat conduction area of the matrix around the spherical filler particles is larger, which is beneficial to participate in the construction of an effective heat conduction channel.

## 4. Electrical and Thermal Properties of Epoxy Resin Composites with Micro-Nano Compound

Compared with micro-particles, nanoparticles have a larger specific surface area, and their particle sizes are small. There is a scale effect at the interface, which can significantly improve the degree of bonding between the particles and the matrix, thereby affecting the electrical and thermal performance of the composite material [46]. Nano particles are small in size, large in surface energy and packed loosely, making it difficult to measure their actual density. Therefore, the proportion of the mass fraction of micro-nano particles is used as a variable in the study of micro-nano composite.

### 4.1. Changes and Analysis of the Breakdown Strength of Composite Materials

**Figure 16:**
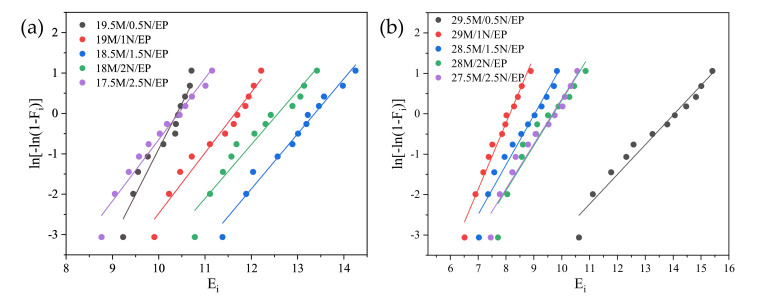
Weibull distribution of micro-nano composite SiC/EP breakdown strength. (**a**) 20 wt% SiC/EP; (**b**) 30 wt% SiC/EP.

By comparing the breakdown voltage of SiC/EP composites with a total filling mass fraction of 20 wt% and 30 wt%, it can be found that under different total filling ratios, the optimal relative ratio of the micro-nano compound has also changed. It can be seen from the current experimental results that for the composite with the total filling mass fraction of 20 wt%, when the ratio of micro and nano was 18.5 wt% and 1.5 wt%, the breakdown strength of the composite material was the highest, and the breakdown strength was increased by 14.9% compared with the case when no nanoparticles were added. For the composite with the total filling mass fraction of 30 wt%, when the ratio of micro and nano was 29.5 wt% and 0.5 wt%, the breakdown strength of the composite material was the highest, and the breakdown strength was increased by 32.4% compared with the case when no nanoparticles were added. Weibull distribution of micro-nano composite SiC/EP breakdown strength is shown in Figure 16.

In micron SiC/EP composites, there will be more air gap defects at the interface between the micron particles and the epoxy resin matrix, which leads to the development of partial discharge channels along the two-phase interface. Thus, the electrical breakdown path of the composite material is formed, and the breakdown strength of the composite material is reduced. After the introduction of nanoparticles, due to the small radius of curvature of the nanoparticles, the groups on the surface of the particles are more likely to react with the epoxy resin instead of bonding with each other [47]. The interface bonding effect between the nanoparticles and the matrix is stronger, and the breakdown path is blocked, so it is unable to develop along the interface [19,48].

**Figure 17:**
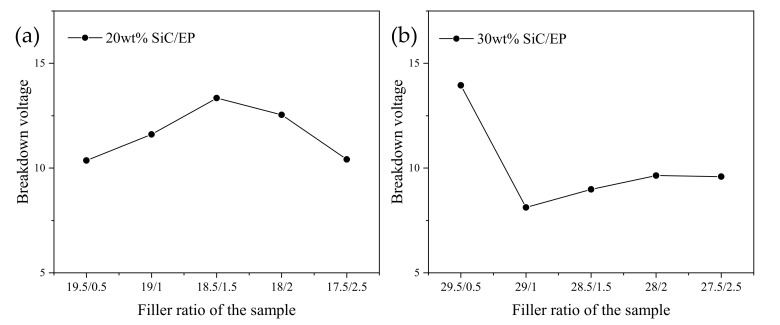
Average breakdown strength of micro-nano composite SiC/EP. (**a**) 20 wt% SiC/EP; (**b**) 30 wt% SiC/EP.

It can be seen from the data in Figure 17 that after adding nano-SiC particles to SiC/EP composites, the breakdown strength of the composites changed with the amount of nano-SiC particles, and there was a certain correspondence with the infrared spectral characteristics of the composites.

**Figure 18:**
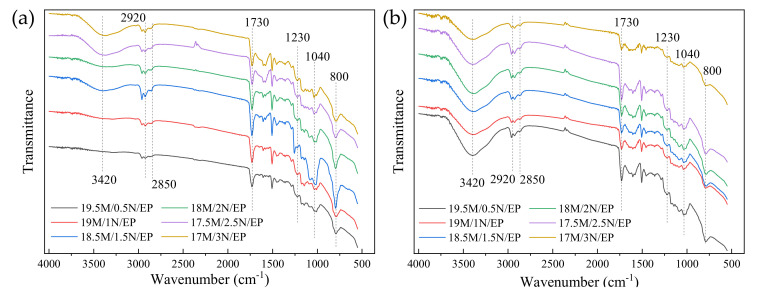
Infrared spectrum of SiC/EP composite material. (**a**) 20 wt% SiC; (**b**) 30 wt% SiC.

**Figure 19:**
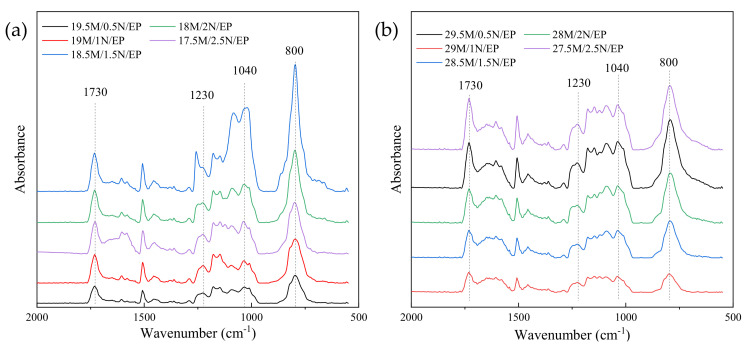
Partial enlarged view of baseline correction of the micro/nano SiC/EP infrared spectra. (**a**) 20 wt% SiC/EP; (**b**) 30 wt% SiC/EP.

The same processing method in Figure 7 was adopted. We converted infrared absorption spectrum into projection spectrum and performed baseline correction and local amplification to visually compare the relative size of each absorption peak, as shown in Figure 18 and Figure 19.

Wavenumber 800 cm^−1^ corresponds to C-N single bond, and the stronger absorption peak represents the greater amide concentration, which indicates that the reaction between tertiary amine promoter and anhydride curing agent was more completed. The reaction rate of tertiary amine accelerator and anhydride curing agent changed when the filling amount of nanoparticles was changed, which also proves that the filling of nanoparticles has a significant effect on the curing reaction of epoxy resin matrix. The wavenumbers of 1230 cm^−1^ and 1170 cm^−1^ are the absorption bands of esters, and the wavenumbers of 1040 cm^−1^ are the absorption bands of primary alcohols. The peak with a wavenumber of 2850cm-1 is caused by the symmetric stretching of C-H in CH3 [24]. The vibrational band at 2920cm-1 is attributed to the vibration of aromatic protons, and the broad peak at 3420cm-1 is hydroxyl and hydrogen bonds [23]. After curing, the higher the crosslinking degree is, the stronger the absorption peaks of corresponding bands are [31,32,33].

When the amount of addition was appropriate, the SiC nanoparticles were uniformly dispersed, whose surface was bonded with the epoxy matrix [19]. The crosslinking degree of the epoxy resin was also improved. However, when the filling amount of nano-SiC particles was too large, the potential energy of the nanoparticle interface will be larger, which will cause agglomeration [49,50]. The equivalent radius of the agglomerate increased, leading to a large number of void defects formed inside, which caused the epoxy cross-linking degree to decrease, and the decrease of breakdown strength of the composite material.

**Figure 20:**
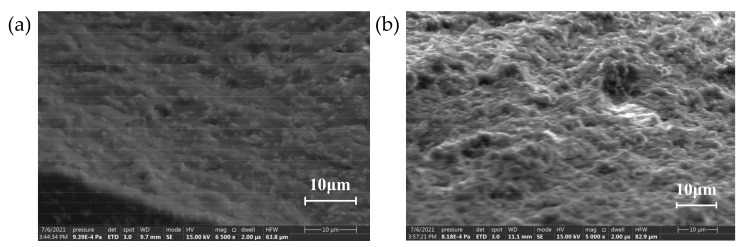
The cross-sectional microscopic electron microscope image of the micro-nano composite system. (**a**) 29.5% m-SiC/0.5%n-SiC/EP; (**b**) 27.5% m-SiC/2.5%n-SiC/EP.

Figure 20 showed that the filler particles are relatively uniformly distributed in the matrix, but when the filling ratio of the nanoparticles exceeded the optimal ratio, the nanoparticles will agglomerate more seriously. The nanoparticles will agglomerate together, and more air gap defects will be formed inside the agglomerated particles. [31] The degree of bonding between the particles and the matrix decreased, resulting in more defects at the cross section. At the same time, in a nano-composite system, when the density of micron SiC particles became larger, the nanoparticles used as reinforcing fillers were more likely to cause agglomeration problems. This was due to the micro- and nanoparticles collide with each other during the mixing process. Since the size of micro-particles and nanoparticles were very different, the movement range of nanoparticles was limited to the gap between the micro-particles, and there was a greater chance for nanoparticles to agglomerate together. Once agglomerating, it was less likely to be separated by the impact force of the stirring process.

### 4.2. Effect of Nanoparticles on Thermal Conductivity of Composites

**Figure 21:**
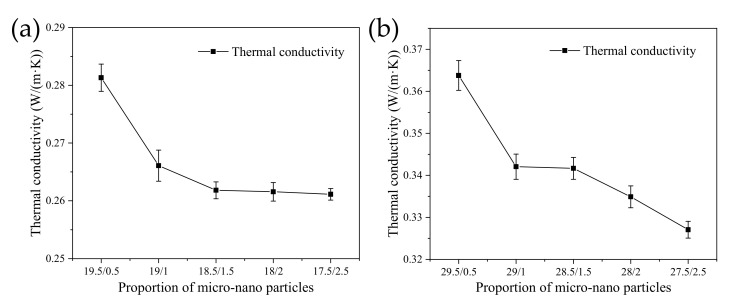
Thermal conductivity of micro-nano compound SiC/EP composite. (**a**) 20 wt% SiC/EP; (**b**) 30 wt% SiC/EP.

When nanoparticles were added to the SiC/EP composite material, the thermal conductivity of composite material changed as shown in the Figure 21:

With the increase of the doping amount of nanoparticles, the thermal conductivity of the composite material decreased. Some previous studies also showed similar results: in the micro-nano composite system, the contribution of nanoparticles to the thermal conductivity is not as good as that of micro-particles [12,39]. This was because nanoparticles were too small in size compared to micro particles. Under the same mass, although the number of nanoparticles was larger, their dispersibility was stronger and they cannot form effective contact similar to larger micron particles, thereby constructing a heat conduction channel. Therefore, as more nanoparticles replaced micron particles, the thermal conductivity of composites showed a downward trend.

## 5. Conclusions

In this paper, in order to improve the electrical and thermal properties of SiC/EP composites, the effects of SiC crystal form and micro nano scale of SiC particles on the breakdown strength and thermal conductivity properties were studied. The main conclusions are as follows:

(1) The compounding of different crystal particles and the compounding of micro-nano particles will significantly affect the bonding degree between the filler particles and the matrix at the interface. For composite materials with different crystal type particles, when the filled particle ratio of α-SiC to β-SiC is 1:1, the breakdown strength of the composite material is more than 10% higher than that of the composite material filled with a single crystal type.

(2) For micro/nano composites, different SiC content corresponds to different optimum ratio of micro to nano. When the amount of SiC increases, the motion space of nanoparticles will be squeezed, which leads to more serious agglomeration and the decrease of corresponding optimal nanometer ratio. Under the optimal ratio, the breakdown strength of the composite material is improved. Compared with the compounding of different crystal types, the introduction of a small amount of nanoparticle can significantly increase the breakdown strength of the micro-nano composite material. 

(3) Whether an effective thermal conductivity channel can be formed is the key to determine the thermal conductivity of composites. The experimental results and the finite element simulation analysis show that the shape and particle size of the filler have a greater impact on the thermal conductivity of the composite. When the shape of the particles is more rounded, the heat conduction channel can be constructed more effectively.

(4) In the filled composite material, the method of compounding different crystalline SiC particles and micro-nano particles can increase the breakdown field strength of the composite, and at the same time make the composite material have a relatively high thermal conductivity, showing certain advantages.

## Figures and Tables

**Table 1:** Information of the raw materials.

Name	Parameter	Manufacturer
E51 type epoxy resin	The epoxy value 0.48~0.54 eq/100 g Viscosity 10~16 Pa·s (25 °C)	Shanghai Xiongrun Resin Co., LTD, Shanghai, China
Methyl tetrahydrophthalic anhydride (curing agent)	Purity > 80% (GC)	TCI Chemical Industrial Development Co., LTD, Toyko, Japan
2,4,6-tris (dimethylaminomethyl) phenol (accelerator)	Purity > 80% (GC)	TCI Chemical Industrial Development Co., LTD, Toyko, Japan
Micron silicon carbide (α,β-SiC)	Particle size: 1.5, 10 µm	Qinhuangdao One nuo Company, Qinhuangdao, China
Nano silicon carbide (n-SiC)	Particle size: 50 nm	Qinhuangdao One nuo Company, Qinhuangdao, China

**Table 2:** SiC/EP composites filled with different crystal forms of SiC particles.

Sample	α-SiC	β-SiC	SiC Volume Fraction
α-SiC/EP_20vol%_	20 vol%	0	20 vol%
3α-SiC/1β-SiC/EP_20vol%_	15 vol%	5 vol%	
1α-SiC/1β-SiC/EP_20vol%_	10 vol%	10 vol%	
1α-SiC/3β-SiC/EP_20vol%_	5 vol%	15 vol%	
β-SiC/EP_20vol%_	0	20 vol%	
α-SiC/EP_30vol%_	30 vol%	0	30 vol%
3α-SiC/1β-SiC/EP_30vol%_	22.5 vol%	7.5 vol%	
1α-SiC/1β-SiC/EP_30vol%_	15 vol%	15 vol%	
1α-SiC/3β-SiC/EP_30vol%_	7.5 vol%	22.5 vol%	
β-SiC/EP_30vol%_	0	30 vol%	

**Table 3:** SiC/EP composites filled with micro/nano particles.

Sample	β-SiC Mass Fraction	n-SiC Mass Fraction	Overall Mass Fraction
19.5M/0.5N/EP	19.5 wt%	0.5 wt%	20 wt%
19M/1N/EP	19 wt%	1 wt%	
18.5M/1.5N/EP	18.5 wt%	1.5 wt%	
18M/2N/EP	18 wt%	2 wt%	
17.5M/2.5N/EP	17.5 wt%	2.5 wt%	
29.5M/0.5N/EP	29.5 wt%	0.5 wt%	30 wt%
29M/1N/EP	29 wt%	1 wt%	
28.5M/1.5N/EP	28.5 wt%	1.5 wt%	
28M/2N/EP	28 wt%	2 wt%	
27.5M/2.5N/EP	27.5 wt%	2.5 wt%	

**Table 4:** The number of filler particles in the RVE geometric model.

Corresponding Crystalline Compound Composite Material	Number of Cube Fillers	Number of Spherical Fillers
α/EP	0	25
3α/1β/EP	7	18
1α/1β/EP	13	12
1α/3β/EP	19	6
β/EP	25	0
